# Malic Acid Supplementation on Rumen Fermentation, Nutrient Digestibility, Performance and Carcass Traits in Lambs: A Meta-Analysis and Meta-Regression Considering Dietary Moderators

**DOI:** 10.3390/ani16081263

**Published:** 2026-04-20

**Authors:** Leonardo Tombesi da Rocha, Fernando Skonieski, Tiago Antonio Del Valle, Francine Basso Facco, Paola de Oliveira Selau, Kamily Pech Oliveira, Amanda de Vasconcelos Zucheto, Julio Viégas

**Affiliations:** 1 Animal Science Department, Federal University of Santa Maria, Avenida Roraima 1000, Santa Maria 97105-900, Brazil; tiago.valle@ufsm.br (T.A.D.V.); francine.facco@acad.ufsm.br (F.B.F.); paolaoliveiraselau@gmail.com (P.d.O.S.); kamily.oliveira@acad.ufsm.br (K.P.O.); amanda.zucheto@acad.ufsm.br (A.d.V.Z.); julio.viegas@ufsm.br (J.V.); 2 Animal Science Department, Federal University of Technology-Parana, Estrada Para Boa Esperança km 04, Dois Vizinhos 85660-000, Brazil

**Keywords:** malate, meta-regression, organic acids, ruminal pH, volatile fatty acids

## Abstract

This meta-analysis and meta-regression evaluated how malic acid supplementation modulates rumen fermentation and its consequences for growth performance, nutrient digestibility, and carcass traits in lambs. Effect sizes (ES) were estimated using a random-effects model. Dietary composition was explored by meta-regression as a key source of heterogeneity, and subgroup analyses were used to compare free malic acid (FMA) and malate. Ruminal pH was not affected by malic acid supplementation. In contrast, total volatile fatty acid concentration increased with malic acid supplementation, particularly in studies using FMA. No effects were detected for propionate concentration, whereas acetate concentration increased (ES = 0.502; *p* = 0.036). A tendency toward a reduced ruminal acetate proportion was observed (ES = −0.683; *p* = 0.072). Malic acid supplementation tended (*p* = 0.057) to increase body weight gain (BWG; ES = 0.325) and final body weight (FBW; ES = 0.234). Malic acid supplementation did not affect carcass traits or overall nutrient digestibility. Meta-regression consistently identified fiber intake-related variables as major moderators of the effects of malic acid. Overall, the effects of malic acid supplementation on lamb performance appear to be primarily driven by its modulation of rumen fermentation and strongly conditioned by dietary context.

## Simple Summary

The restriction of antimicrobial feed additives has increased interest in nutritional strategies that can support rumen function and animal performance. Malic acid is a naturally occurring organic acid that has been proposed as a dietary supplement for ruminants because of its potential to influence ruminal fermentation. However, results from studies conducted with lambs are inconsistent, limiting clear conclusions about its effectiveness. This study combined data from multiple experiments to evaluate the effects of malic acid supplementation on ruminal fermentation, growth performance, carcass traits, and nutrient digestibility in lambs, while accounting for differences in diet composition. The results showed that malic acid supplementation increased the concentration of fermentation products that supply energy to the animal, particularly in diets with higher forage inclusion. Small improvements in growth performance were observed, whereas carcass characteristics and overall nutrient digestibility were not consistently affected. The findings indicate that the response to malic acid supplementation depends strongly on dietary conditions rather than on the additive alone. These results help explain the variability reported in individual studies and highlight the importance of considering diet composition when evaluating organic acid supplementation strategies in lamb production.

## 1. Introduction

Growing public concern about food quality and the emergence of bacterial resistance to antibiotics in animal production have driven the search for effective alternatives to conventional antimicrobial additives. Although no documented cases link ionophores to bacterial resistance, these compounds have been regulated by the European Union and classified as prohibited feed additives since 2009, further reinforcing the need for alternative nutritional strategies [1]. Within this context, increasing attention has been directed toward dietary approaches capable of modulating rumen fermentation without relying on antimicrobial mechanisms.

Among the proposed alternatives, malic acid, an organic acid naturally present in several feedstuffs and directly involved in the metabolic pathways of ruminal microorganisms, has emerged as a promising option. Its primary mode of action involves stimulation of lactate utilization, particularly by *Selenomonas ruminantium*, contributing to increased ruminal pH and enhanced propionate production [2,3]. Based on these mechanisms, malic acid supplementation is expected to increase growth performance in lambs, since propionate is the major glucogenic volatile fatty acid, directly contributing to hepatic gluconeogenesis and energy supply in ruminants [4]. However, in vivo evidence remains inconsistent, precluding definitive conclusions regarding its efficacy as a dietary additive for lambs [5,6,7,8].

Over the years, researchers have sought to identify the sources of these inconsistencies by evaluating several factors, including differences in diet composition [9], supplementation dose [10], and the chemical form of malic acid (free acid vs. malate salt). Notably, many of these factors are intrinsically linked to the ruminal fermentation environment, suggesting that responses to malic acid supplementation may be strongly context-dependent rather than additive-specific. Beyond these biological sources of variation, statistical limitations such as insufficient power in individual experiments may also contribute to the variability in reported results [11].

In this context, meta-analysis combined with meta-regression can be used to synthesize the available evidence on malic acid supplementation in lambs, while accounting for potential dietary moderators [12]. This approach allows the quantification of overall effects and the exploration of sources of heterogeneity across studies, helping to identify response variables influenced by malic acid as well as the key factors driving or modulating these responses, an aspect that remains poorly addressed in the current literature.

Therefore, the objective of this study was to quantitatively evaluate how malic acid supplementation modulates rumen fermentation and its associated effects on growth performance, carcass characteristics, and nutrient digestibility in lambs, using meta-analysis and meta-regression to identify dietary factors driving heterogeneity in the observed responses.

## 2. Materials and Methods

The database was composed of peer-reviewed published studies evaluating the effects of malic acid supplementation on performance, carcass characteristics, nutrient digestibility and rumen parameters of lambs. The literature search was conducted using the Web of Science, PubMed, and Google Scholar databases, applying the following keywords: malic acid, malate, sheep, and lambs. In addition, the reference lists of the selected studies were screened to identify further relevant publications.

Studies included in the meta-analysis were required to be original research articles reporting a mean value and a measure of dispersion for the variables of interest. When standard deviations were not explicitly provided, they were calculated from the variability measures reported in the original studies, following the procedures recommended in the Cochrane Handbook for Systematic Reviews of Interventions [12]. Only studies presenting results for both a control group and at least one treatment group (free malic acid and/or malate) were considered eligible. Independent experiments reported within the same article were treated as separate comparisons.

When multiple doses of malic acid were evaluated within the same experiment, each dose was treated as an independent comparison using a split-control approach. To prevent artificial inflation of the control group weight and violation of the assumption of independence, the sample size of the shared control group was proportionally divided among comparisons, following the methodological recommendations of Higgins et al. [12].

The information included was related to study, periodical, year, design, dose, forage:concentrate ratio, chemical composition of the feed, breed, initial weight, among others. If not present in the articles, the composition of the diets was calculated (Table 1). Therefore, only studies containing the chemical composition of diets or that provided information that allowed it to be estimated were included. The variables of interest included in the analysis were those related to performance, carcass characteristics, digestibility of dietary fractions, and rumen parameters, totaling 21 variables. The final version of the database contains 12 articles that allowed up to 19 comparisons.

The effect of malic acid supplementation was evaluated using the standardized mean difference (SMD), calculated as Hedges’ g, which includes a small-sample bias correction to Cohen’s d [12]. Effect sizes were computed as the difference between the malic acid and control groups divided by the pooled standard deviation and subsequently corrected for bias. The overall effect of malic acid supplementation was estimated using a random-effects model, with between-study variance (τ2) estimated by restricted maximum likelihood (REML). Between-study heterogeneity was assessed using Cochran’s Q test, and the I^2^ statistic was used to quantify the proportion of total variability attributable to heterogeneity rather than sampling error [12]. Effect estimates are reported with 95% confidence intervals.

Meta-regression analyses were conducted using a random-effects model to explore covariates as potential sources of variability in responses to malic acid supplementation. Although a minimum of 10 comparisons is generally recommended for meta-regression, analyses were performed for all variables that exhibited at least low to moderate heterogeneity (I^2^ > 25%). Meta-regressions based on fewer than 10 comparisons should therefore be interpreted as exploratory in nature. The *t* test described by Knapp and Hartung [19] was used instead of the traditional z test in order to reduce the likelihood of false-positive results given the low number of comparisons. The R^2^ analog was used to quantify the proportion of between-study heterogeneity explained by the meta-regression model [20].

The following explanatory variables were evaluated: forage intake, concentrate intake, forage proportion (forage intake/(forage intake + concentrate intake), crude protein intake, neutral detergent fiber intake, acid detergent fiber intake, starch intake, ether extract intake, and malic acid dose, expressed both as g/day and g/kg body weight (BW). Variables expressed in g/day were analyzed but are not presented, as expressions relative to BW (g/kg BW) yielded more consistent and interpretable results across studies. For the dose covariate, only the amount of commercial malic acid product or free acid reported by the authors was considered; potential contributions of malic acid naturally present in feed ingredients were not accounted for. Results for malic acid dose (g/kg BW) and ether extract intake (g/kg BW) were omitted from the final version of the manuscript because they did not show statistically significant associations.

A subgroup analysis was performed to evaluate whether the chemical form of malic acid supplementation (free malic acid or malate) influenced the magnitude and direction of the observed effects, with the purpose of exploring form-specific responses and potential sources of between-study heterogeneity. For some variables, the number of comparisons within the free malic acid (FMA) subgroup was limited to only two studies; therefore, results for these subgroups should be interpreted as exploratory. In addition, for certain variables, no studies evaluating the free acid form were available; in such cases, the overall effect represents exclusively the effect of malic acid supplementation in the salt form (malate).

Risk-of-bias (RoB) assessment was conducted for 12 primary studies using a prespecified tool adapted from the recommendations of the Cochrane Collaboration for randomized and non-randomized studies, in order to reflect the specific characteristics of animal production research. This adaptation was based on evidence that tools originally developed for human health research may not be directly applicable to animal studies, particularly due to limitations in the reporting of procedures such as randomization [21]. The tool comprised five domains: bias arising from the randomization process, bias due to deviations from intended interventions, bias due to missing outcome data, bias in outcome measurement, and bias in the selection of reported results. Judgments were classified as low risk of bias, some concerns, or high risk of bias.

Specific criteria were defined to standardize the assessment. Studies using a completely randomized design were classified as low risk in the randomization domain when random allocation of animals was reported, as this is standard practice in animal production studies. For studies using block designs, the absence of information regarding block formation criteria, number of blocks, or range of variation among animals (e.g., body weight) resulted in a classification of “some concerns”. In the domain of missing data, any report of mortality, loss, or removal of animals led to a classification of “some concerns”. For outcome measurement, blinding was not considered, as it is not commonly applied in animal production studies; however, incomplete or inadequately described measurement techniques were classified as “some concerns”. Summary plots were generated using the Robvis R Package (version 0.3.0) [22] with a generic template.

The leave-one-out analysis was conducted as a sensitivity test to evaluate the influence of individual studies on the overall effect estimates by sequentially removing one study at a time. Publication bias was assessed by visual inspection of funnel plots to examine potential asymmetry suggestive of small-study effects. All statistical analyses were performed using Stata software (version 17).

## 3. Results

### 3.1. Risk of Bias

**Figure 1:**
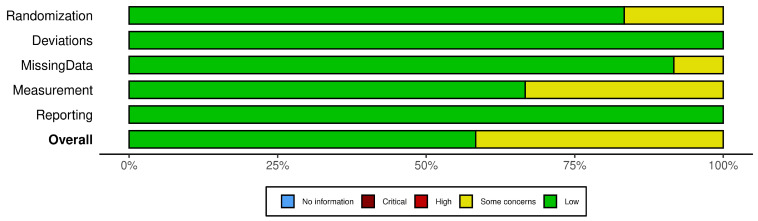
Distribution of risk-of-bias judgments within each bias domain for the 12 studies investigating the effects of malic acid in lambs.

Of the 12 studies assessed, seven were classified as low risk of bias and five as having some concerns, with no studies classified as high risk. The main limitations were associated with the randomization domain, particularly in studies using block designs without adequate methodological description, as well as in the reporting of outcome measurement procedures. The distribution of risk-of-bias judgments across domains is presented in Figure 1. Study-level risk-of-bias assessments are provided in the Supplementary Material (Figure S1).

### 3.2. Ruminal Parameters

The meta-analysis of 12 effect sizes indicated that malic acid supplementation had no significant effect on ruminal pH (ES = 0.117 [−0.174; 0.408]; *p* = 0.432), with no evidence of heterogeneity (I^2^ = 0%; *p* = 0.672) (Table 2). Similarly, no overall effect was observed for NH_3_-N (ES = −0.531 [−1.211; 0.149]; *p* = 0.127); however, heterogeneity was substantial and statistically significant (I^2^ = 69%; *p* = 0.003). The leave-one-out analysis for NH_3_-N (Figure S2) indicated that no single study exerted an undue influence on the overall effect estimate. Although visual inspection of the funnel plot suggested some asymmetry (Figure S3), this pattern should be interpreted in the context of the substantial heterogeneity observed. Meta-regression analyses identified forage intake (slope = −0.112; *p* = 0.006; R^2^ = 97.82%) and the forage proportion (slope = −4.816; *p* = 0.005; R^2^ = 100%) as significant moderators of the effect size.

Total VFA concentration (NC = 10) was significantly higher in the malic acid group (ES = 0.558 [0.155; 0.960]; *p* = 0.007), with non-significant but low-to-moderate between-study heterogeneity (*p* = 0.144; I^2^ = 29). There was a difference between subgroups (*p* = 0.01); although both indicated the same direction of effect, the effect size of FMA on total VFA concentration was almost six times greater than that observed for the malate group (1.277 vs. 0.222). The leave-one-out analysis indicated that no study included in the meta-analysis was influential, as the result remained significant across all iterations (Figure S4). The meta-regression identified that forage intake (slope = 0.066; *p* = 0.033; R^2^ = 100%) and the forage proportion (slope = 2.295; *p* = 0.047; R^2^ = 83.08%) explained all the heterogeneity associated with the model.

The addition of malic acid increased acetate concentration (ES = 0.474; *p* = 0.047), whereas no effects were observed on propionate (ES = 0.239; *p* = 0.309) or butyrate concentrations (ES = 0.299; *p* = 0.205). There was no evidence of heterogeneity associated with these variables (I^2^ = 0; *p* > 0.05). The leave-one-out analysis showed that the overall effect was sensitive to the exclusion of individual studies. For the propionate and butyrate concentrations, the *p*-value remained non-significant regardless of which study was removed (Figure S4). Subgroup analysis was not performed because no studies were found that used FMA and measured the individual concentration of these VFAs.

The meta-analysis showed that malic acid supplementation had no significant effect on ruminal VFA proportions in lambs (NC = 6). The acetate proportion showed a tendency to decrease (ES = −0.683 [−1.427; 0.062]; *p* = 0.072), with substantial and significant heterogeneity (I^2^ = 68; *p* = 0.008). No significant effects were observed for propionate (ES = 0.371 [−0.333; 1.075]; *p* = 0.302) or butyrate proportions (ES = 0.365 [−0.236; 0.966]; *p* = 0.234), both associated with substantial heterogeneity (I^2^ = 64 and 53, respectively). Likewise, the acetate-to-propionate ratio was not affected by malic acid supplementation (ES = −0.269 [−0.724; 0.186]; *p* = 0.247), with low and non-significant heterogeneity (I^2^ = 22; *p* = 0.321). None of the tested covariates was able to explain the heterogeneity associated with the effect of malic acid on acetate proportion. For propionate proportion, three fiber intake-related covariates were significant: ADF intake (slope = 0.272; *p* = 0.029; R^2^ = 100%), forage intake (slope = 0.192; *p* = 0.041; R^2^ = 100%), and the forage proportion (slope = 6.944; *p* = 0.043; R^2^ = 85.19%). Regarding butyrate proportion, although an R^2^ of 100% was observed for the moderators’ CP intake, starch intake, and concentrate intake, none of the corresponding coefficients were significant according to the *t*-test.

Because no studies evaluated the free acid form for individual VFA proportion, the overall effects exclusively reflect results derived from malate supplementation. The leave-one-out analysis for acetate proportion showed that removal of the study by Gonzalez-Momita et al. [13] resulted in a significant effect size (ES = −0.860; *p* = 0.041). For propionate and butyrate proportions, as well as for the acetate-to-propionate ratio, the *p*-value remained non-significant regardless of which study was removed (Figures S6 and S7).

### 3.3. Nutrient Digestibility

No significant effects (*p* > 0.05) of malic acid supplementation were observed on the digestibility of dry matter (DMDIG), organic matter (OMDIG), crude protein (CPDIG), neutral detergent fiber (NDFDIG), or acid detergent fiber (ADFDIG) (Table 3). A significant subgroup effect (FMA vs. malate) was detected for ADFDIG (*p* < 0.01). The direction and magnitude of the effect on ADFDIG in animals receiving FMA (ES = 1.344; *p* = 0.003) were opposite to and more than fivefold greater than those observed in animals supplemented with malate (ES = −0.255; *p* = 0.340). Leave-one-out analyses for nutrient digestibility confirmed that no individual study exerted undue influence, as the overall effects remained non-significant across all iterations (Figure S8). The apparent asymmetry in the funnel plots (Figure S9) should be interpreted in the context of the moderate to high heterogeneity associated with these variables.

All variables exhibited significant heterogeneity (*p* < 0.05), of moderate to high magnitude, with I^2^ values ranging from 30 to 73. None of the covariates significantly explained the heterogeneity associated with DMDIG and CPDIG. In contrast, for OMDIG, positive slope coefficients were observed for NDF intake (slope = 0.239; *p* = 0.037; R^2^ = 43.11) and ADF intake (slope = 0.276; *p* = 0.016; R^2^ = 60.64), whereas concentrate intake showed a negative coefficient (slope = −0.037; *p* = 0.050; R^2^ = 53.53). Similarly, more than one covariate significantly explained the heterogeneity associated with ADFDIG. Positive slope coefficients were observed for forage intake (slope = 0.085; *p* = 0.029; R^2^ = 77.20%) and the forage proportion (slope = 3.936; *p* = 0.020; R^2^ = 84.96%), whereas negative coefficients were observed for protein intake (slope = −0.282; *p* = 0.008; R^2^ = 100%) and concentrate intake (slope = −0.041; *p* = 0.010; R^2^ = 98.54%). For NDFDIG, forage intake (slope = 0.053; *p* = 0.049; R^2^ = 100%) and the forage proportion (slope = 2.300; *p* = 0.049; R^2^ = 100%) were significant.

### 3.4. Growth Performance and Carcass Characteristics

No effect of malic acid supplementation was detected on dry matter intake (DMI; NC = 17) and feed conversion ratio (FCR; NC = 12) (Table 4). Leave-one-out analyses for DMI (Figure S10) confirmed that no individual study exerted undue influence, as the overall effects remained non-significant and stable across all iterations. In addition, visual inspection of the funnel plot (Figure S11) did not indicate marked asymmetry, suggesting no clear evidence of publication bias for DMI. For these variables, no significant subgroup effects were observed when studies were stratified according to malic acid form (FMA vs. malate).

A trend toward increased body weight gain (BWG; NC = 16) was observed in animals supplemented with malic acid (0.325 [−0.010; 0.661]; *p* = 0.057). No subgroup effect was detected (*p* = 0.120); however, the estimated effect size (ES) for FMA was approximately eight times greater than that observed for the malate subgroup (Table 4). Overall heterogeneity was significant, although of low to moderate magnitude (I^2^ = 37; *p* = 0.029). The leave-one-out analysis indicated high sensitivity of the overall effect estimate to the exclusion of individual studies (Figure S10). Meta-regression showed that forage intake (slope = 0.071; *p* = 0.019; R^2^ = 46.56), concentrate intake (slope = −0.018; *p* = 0.046; R^2^ = 59.88), and the forage proportion (slope = 3.863; *p* = 0.004; R^2^ = 87.44) were significant covariates, explaining total or partial residual heterogeneity in the model (Table 5).

The meta-analysis based on 18 comparisons showed a trend (*p* = 0.057) toward increased final body weight (FBW) in lambs receiving malic acid supplementation. A significant subgroup effect was observed (*p* = 0.042), with FMA showing a significant and greater effect (ES = 0.753 [0.162; 1.343]; *p* = 0.013) compared with malate, which did not show a significant effect (ES = 0.061 [−0.223; 0.474]; *p* = 0.657). The overall effect estimate for this variable was sensitive to the exclusion of individual studies, with statistical significance ranging from non-significant (*p* > 0.10) to significant (*p* < 0.05) (Figure S10).

Carcass characteristics, including hot carcass weight (HCW; NC = 9) and carcass yield (CY; NC = 9), were unaffected by malic acid supplementation (Table 4). No significant subgroup effects were detected for carcass characteristics. Overall heterogeneity was not statistically significant for any carcass variable (*p* > 0.05), with I^2^ values close to zero in most cases.

## 4. Discussion

### 4.1. Ruminal Parameters

The present meta-analysis did not provide evidence that malic acid supplementation alters ruminal pH in lambs under in vivo conditions. The overall estimated effect size (ES = 0.117), combined with the absence of heterogeneity both overall and within subgroups (I^2^ = 0%), indicates that the small numerical differences observed among studies are consistent with random variation rather than a biologically meaningful effect [11]. In line with this interpretation, the similarity of effect sizes between FMA (ES = 0.145) and malate (ES = 0.106) further supports the lack of a differential or treatment-specific response. This result contrasts with the consistent pH-stabilizing effects reported in in vitro systems, which were a major driver for proposing malic acid as a potential alternative to ionophores [23]. However, the mechanisms underlying pH modulation differ substantially between these additives. Whereas ionophores primarily act by inhibiting lactate-producing bacteria, malic acid exerts its effect indirectly by stimulating lactate-utilizing bacteria such as *Selenomonas ruminantium* [24,25]. The lack of a detectable in vivo effect on pH may therefore reflect the lower microbial densities, greater substrate complexity, and higher buffering capacity of the rumen environment compared with controlled in vitro systems [26]. A critical limitation identified in the available literature is the near absence of direct measurements of ruminal lactate concentration. Among the studies included in this meta-analysis, only Carro et al. [10] reported lactate data. Given that the hypothesized mode of action of malic acid relies on enhanced lactate uptake, the routine inclusion of ruminal lactate measurements in future in vivo studies is essential to link malic acid supplementation with observed fermentative results.

The effect of malic acid supplementation on ruminal ammonia nitrogen (NH_3_-N) also remained inconclusive. Although the overall effect size was negative (ES = −0.531), indicating a tendency toward lower NH_3_-N concentrations, the effect did not reach statistical significance and was associated with substantial heterogeneity (I^2^ = 69%). The marked difference in effect size magnitude between the FMA (ES = −1.237) and malate (ES = −0.081) subgroups suggests that variability in responses may be driven by contextual factors rather than by random error alone. Free malic acid (FMA) corresponds to the protonated form of the molecule, whereas malate is its dissociated form, typically supplied as a salt (e.g., calcium malate). Studies conducted in beef cattle suggest that there are no significant differences between these chemical forms [27,28], and no studies in lambs have directly compared FMA and malate within the same experiment. Therefore, the greater response observed for FMA in the present analysis is unlikely to reflect an intrinsic effect of chemical form, but rather differences in the dietary conditions under which each form was evaluated. Ruminal NH_3_-N concentration reflects the balance between nitrogen release and its incorporation into microbial protein, which is primarily governed by the temporal synchronization between fermentable carbohydrate availability and ammonia release, rather than forage inclusion per se [29]. In this context, diets combining forage and concentrate typically promote greater microbial protein synthesis than diets dominated by a single component [30]. Consistently, the negative regression coefficients observed for forage intake and forage proportion indicate that diets with greater forage inclusion tend to exhibit larger reductions in NH_3_-N in response to malic acid supplementation. Notably, the maximum forage proportion in the database was only 0.50, which may limit extrapolation to higher-forage systems. It is important to note that high forage proportions may also introduce confounding, as forages, particularly legumes, contain intrinsic malic acid that can contribute substantial background levels of malate to the rumen [31]. This endogenous supply may reduce the relative contribution of supplemental malic acid, attenuating the observed response and potentially leading to a plateau effect that limits detectable differences between treatment and control groups.

Total ruminal volatile fatty acid (VFA) concentration was the most consistent fermentative response to malic acid supplementation, with a significant overall increase (ES = 0.558). The subgroup analysis revealed a markedly greater effect associated with FMA compared with malate (1.277 vs. 0.222), although both forms showed effects in the same direction. Malate has been proposed to stimulate propionate production via the succinate–propionate pathway, involving dehydration of malate to fumarate, reduction to succinate, and subsequent conversion to propionate, a pathway closely associated with *S. ruminantium* [3]. Nevertheless, the end products of this metabolism include not only propionate but also succinate and acetate [31]. Meta-regression analyses consistently identified forage intake and the forage proportion as key moderators, indicating that higher forage inclusion was associated with a greater magnitude of response to malic acid supplementation. Importantly, this does not imply that absolute VFA concentrations were higher in high-forage diets, but rather that the difference between supplemented and control groups was larger under these conditions. A plausible explanation is that the high concentrate inclusion observed in most studies limited the scope for further enhancement of fermentation, as propionate-oriented fermentation was already strongly established. However, it is important to acknowledge that, although malic acid clearly modulates rumen fermentation end-products such as VFAs, the mechanistic interpretation of these responses remains limited by the type of data available in the primary studies. None of the studies included in this meta-analysis reported microbial community composition, functional gene expression, or metabolomic profiles, which would be necessary to further elucidate the underlying microbiological mechanisms driving these fermentative shifts.

Despite the increase in total VFA concentration, malic acid supplementation did not consistently affect individual propionate or butyrate concentrations, while acetate concentration increased significantly. This pattern is mechanistically plausible. When the capacity of the succinate–propionate pathway is exceeded, acetate becomes a secondary end product of malate metabolism in *S. ruminantium* [31]. In highly fermentable diets characterized by rapid substrate availability, carbon flow may be redirected toward acetate rather than propionate [32,33]. Given that the average concentrate inclusion across studies was 88.7%, this fermentative context likely constrained the detectable effect on propionate concentration relative to acetate. As expected, no effect on butyrate concentration was observed, as no direct metabolic pathway linking malic acid fermentation to butyrate production has been described [3].

Regarding VFA proportions, the tendency toward a reduced acetate proportion (ES = −0.683; *p* = 0.072) aligns with the role of acetate as a secondary end product of malate metabolism. However, the absence of a significant effect on propionate proportion, butyrate proportion, or acetate-to-propionate ratio reflects both the modest magnitude of these effects and the substantial heterogeneity observed [34]. Meta-regression suggested that fiber-related variables positively modulate the effect of malic acid on propionate proportion, reinforcing the concept that malic acid exerts more pronounced effects under diets with a more balanced forage proportion. Similar diet-dependent responses have been described for ionophores, despite fundamental differences in their mechanisms of action [25,35,36]. Given the limited number of comparisons, these results should be interpreted as hypothesis-generating.

### 4.2. Nutrient Digestibility

The present meta-analysis did not identify consistent effects of malic acid supplementation on the digestibility of dry matter, organic matter, crude protein, neutral detergent fiber, or acid detergent fiber. These results are consistent with the variability reported in individual in vivo studies [9,14,15] and highlight the difficulty of extrapolating in vitro responses to whole-animal conditions. Although malic acid has been shown to enhance organic matter disappearance in vitro by stabilizing ruminal pH and stimulating lactate utilization [24], these mechanisms appear to be strongly modulated by dietary context in vivo.

A significant subgroup effect was detected for acid detergent fiber digestibility, favoring FMA supplementation. However, this result is based on a limited number of comparisons and should be interpreted cautiously. Nonetheless, the consistent tendency toward positive effect sizes for FMA across several digestibility variables suggests that malic acid may enhance fiber utilization under specific fermentative conditions rather than exerting a generalized effect.

Meta-regression analyses revealed a coherent pattern in which fiber intake–related variables and the forage proportion were positively associated with malic acid effects, whereas concentrate intake showed negative associations. This pattern supports the hypothesis that malic acid supplementation is more effective in diets with greater structural carbohydrate availability, where fermentation is less dominated by rapidly fermentable substrates. In contrast, in high-concentrate diets, lactate production may exceed the metabolic capacity of lactate-utilizing bacteria, thereby limiting the potential benefits of malic acid on digestibility [37]. Protein intake emerged as a significant moderator for ADF digestibility, likely reflecting indirect associations with concentrate inclusion and overall dietary fermentability rather than a direct causal relationship.

### 4.3. Performance and Carcass Characteristics

Malic acid supplementation did not affect dry matter intake (DMI) in lambs, as evidenced by the near-zero overall effect size (ES = −0.002) and complete overlap between treatment and control group means. Although reductions in DMI have been reported in dairy cows and attributed to palatability issues [38], such effects were not evident in lambs. The lack of effect is consistent with a recent meta-analysis showing no impact of fumaric acid on feed intake in small ruminants [39], suggesting that organic acids do not exert a primary regulatory role on intake under the dietary conditions evaluated.

The observed trend toward increased body weight gain (BWG; ES = 0.325; *p* = 0.057) is biologically plausible and aligns with the documented increase in ruminal VFA concentration. VFAs are directly linked to ruminal epithelial development and host energy supply [4,40]. However, the moderate heterogeneity observed (I^2^ = 37%) and the higher heterogeneity within the acid subgroup (I^2^ = 66%) indicate that performance responses are not uniform and likely represent secondary consequences of underlying fermentative changes rather than direct effects of the additive.

Meta-regression analyses consistently identified the forage proportion, forage intake, and concentrate intake as key moderators of BWG response. Although malic acid is mechanistically associated with increased propionate production via lactate uptake [24], the predominance of high-concentrate diets in the database (mean forage proportion = 0.145; five comparisons >90% concentrate) likely limited the potential for additional improvement. In such contexts, fermentation may already be maximally oriented toward propionate production, as observed for ionophores [36].

No significant effect of malic acid supplementation on feed conversion ratio (FCR) was detected. Although numerical differences were observed across individual studies [5,7,8], the small overall effect size (ES = −0.170) and absence of heterogeneity (I^2^ = 0) suggest that these differences are largely attributable to sampling error. Nonetheless, small effect sizes may still be biologically relevant depending on the outcome assessed [41]. Under an assumed FCR of 4 kg/kg with an SD of 0.5, the observed ES corresponds to a reduction of 0.085 units, which may be economically meaningful under certain production systems.

A trend toward increased final body weight (FBW) was observed, reinforcing the findings for BWG. Subgroup analysis indicated a greater effect for FMA (ES = 0.753; *p* = 0.013), whereas malate showed no effect. Importantly, no studies directly compared the two chemical forms within the same experimental design, and the moderate heterogeneity observed within the FMA subgroup (I^2^ = 36%) suggests that these differences should not be interpreted as evidence of intrinsic superiority. Exploratory meta-regression identified concentrate intake as a significant moderator, supporting the hypothesis that dietary context, rather than chemical form per se, drives the observed differences.

Finally, malic acid supplementation did not affect carcass characteristics, including hot carcass weight and carcass yield. The small, non-significant effect sizes indicate no statistical evidence for population-level effects [42]. Nonetheless, these estimates are valuable for informing sample size calculations in future studies [43]. The low number of comparisons within the acid subgroup precluded further exploration of heterogeneity, in accordance with recommendations to avoid spurious findings in underpowered meta-regressions [44].

## 5. Conclusions

Malic acid supplementation modulates rumen fermentation in lambs, with its effects being strongly conditioned by dietary context. The present meta-analysis and meta-regression indicate that increases in volatile fatty acid concentration, particularly under diets with higher forage inclusion and more balanced forage proportion, represent the most consistent fermentative response to malic acid supplementation.

Improvements in growth performance appear to be consequences of these fermentative changes rather than direct effects of the additive itself, which helps explain the inconsistent responses reported in individual in vivo studies. Differences observed between free malic acid and malate should be interpreted with caution, as available evidence suggests that dietary characteristics play a more significant role than the chemical form itself.

Overall, the results highlight the importance of considering the rumen fermentation environment when evaluating organic acid supplementation strategies. Future studies should prioritize integrated assessments of ruminal fermentation dynamics, including lactate metabolism and microbial activity, under well-characterized dietary conditions, to better elucidate the mechanisms through which malic acid influences ruminant productivity.

## Figures and Tables

**Table 1:** Chemical form of malic acid, main cereal and forage sources, supplement dose, and total mixed ration composition in experiments with lambs.

Paper	Form	Main Cereal	Main Forage	CP %	NDF %	ADF %	Starch %	EE %	Dose g/Day	Forage Proportion ^1^
Heredia et al. [5] a	Malate	Corn	Hay	17.20	21.40	14.70	43.14	4.80	2.92	0.16
Heredia et al. [5] b	Malate	Corn	Hay	17.20	21.40	14.70	43.14	4.80	2.97	0.16
Ali et al. [6] a	FMA	Barley	None	15.07	22.19	5.31	38.13	2.62	1.14	0.00
Ali et al. [6] b	FMA	Barley	None	15.07	22.19	5.31	38.13	2.62	2.27	0.00
Elewa et al. [7] a	FMA	Corn	Silage	13.60	33.50	19.40	40.70	2.50	5.14	0.50
Elewa et al. [7] b	FMA	Corn	Silage	13.60	33.50	19.40	40.70	2.50	10.29	0.48
Heredia et al. [8]	Malate	Corn	Straw	14.95	15.52	6.62	44.01	3.92	3.28	0.15
Mungói et al. [9] a	Malate	Wheat	Straw	18.43	13.57	5.45	42.60	2.77	1.93	0.07
Mungói et al. [9] b	Malate	Barley	Straw	17.05	14.04	5.72	44.13	3.81	2.05	0.04
Carro et al. [10] a	Malate	Barley	Straw	15.51	15.01	4.95	50.13	2.56	3.37	0.07
Carro et al. [10] b	Malate	Barley	Straw	15.49	14.99	4.95	50.07	2.56	7.31	0.06
Gonzalez-Momita et al. [13]	Malate	Sorghum	None	15.70	19.30	8.28	48.61	2.79	2.11	0.00
Elmali et al. [14] a	FMA	Barley	Hay	14.48	30.28	13.27	33.23	3.19	3.92	0.17
Elmali et al. [14] b	FMA	Barley	Hay	14.48	30.28	13.27	33.23	3.19	7.84	0.17
Malekkhahi et al. [15]	Malate	Barley	Silage	15.00	36.00	27.00	32.00	3.00	3.54	0.30
Loya-Olguin et al. [16]	FMA	Sorghum	Straw	14.01	16.44	5.76	44.36	3.88	3.93	0.07
Toprak et al. [17] a	Malate	Barley	Hay	17.67	27.50	12.15	49.30	3.31	4.43	0.07
Toprak et al. [17] b	Malate	Barley	Hay	17.36	28.96	11.87	45.96	3.09	9.44	0.09
Yarahmadi et al. [18]	Malate	Corn	Hay	16.77	16.08	9.70	40.39	2.74	15.00	0.20

CP, Crude Protein; NDF, Neutral detergent fiber; ADF, Acid detergent fiber; EE, Ether extract; ^1^ Forage proportion was calculated as forage/(forage + concentrate), based on the actual intake values reported in the studies; Lowercase letters (a, b) indicate different comparisons within the same study.

**Table 2:** Summary of meta-analysis results for the effects of malic acid supplementation on ruminal parameters and volatile fatty acid concentration and proportion in lambs.

Variable	Subgroup	NC	ES [CI]	ES *p*-Value	I^2^	Het *p*-Value
pH	FMA	4	0.145 [−0.411; 0.701]	0.609	0	0.992
	Malate	8	0.106 [−0.236; 0.448]	0.543	0	0.303
	Overall	12	0.117 [−0.174; 0.408]	0.432	0	0.672
Ammonia nitrogen	FMA	4	−1.237 [−2.737; 0.264]	0.106	81	0.001
	Malate	5	−0.081 [−0.541; 0.379]	0.730	0	0.962
	Overall	9	−0.531 [−1.211; 0.149]	0.127	69	0.003
VFA concentration						
Total VFA	FMA	4	1.277 [0.538; 2.016]	0.001	28	0.238
	Malate	6	0.222 [−0.171; 0.615]	0.268	0	0.935
	Overall *	10	0.558 [0.155; 0.960]	0.007	29	0.144
Acetate	FMA					
	Malate	4	0.502 [0.033; 0.970]	0.036	0	0.563
	Overall	4	0.502 [0.033; 0.970]	0.037	0	0.563
Propionate	FMA					
	Malate	4	0.239 [−0.221; 0.699]	0.309	0	0.817
	Overall	4	0.239 [−0.221; 0.699]	0.309	0	0.817
Butyrate	FMA					
	Malate	4	0.299 [−0.163; 0.761]	0.205	0	0.717
	Overall	4	0.299 [−0.163; 0.761]	0.205	0	0.717
VFA proportion						
Acetate	FMA					
	Malate	6	−0.683 [−1.427; 0.062]	0.072	68	0.008
	Overall	6	−0.683 [−1.427; 0.062]	0.072	68	0.008
Propionate	FMA					
	Malate	6	0.371 [−0.333; 1.075]	0.302	64	0.025
	Overall	6	0.371 [−0.333; 1.075]	0.302	64	0.025
Butyrate	FMA					
	Malate	6	0.365 [−0.236; 0.966]	0.234	53	0.063
	Overall	6	0.365 [−0.236; 0.966]	0.234	53	0.063
Acetate:propionate	FMA					
	Malate	6	−0.269 [−0.724; 0.186]	0.247	22	0.321
	Overall	6	−0.269 [−0.724; 0.186]	0.247	22	0.321

NC, Number of comparisons; ES, effect size; CI confidence interval; Het, heterogeneity; FMA, free malic acid; *, *p* < 0.05 for between-group differences (Q_b test; χ^2^).

**Table 3:** Summary of meta-analysis results for the effects of malic acid supplementation on lamb nutrient digestibility.

Variable	Subgroup	NC	ES [CI]	ES *p*-Value	I^2^	Het *p*-Value
Dry matter	FMA	4	0.563 [−0.207; 1.332]	0.152	42	0.162
	Malate	5	−0.110 [−0.758; 0.538]	0.739	54	0.072
	Overall	9	0.165 [−0.355; 0.685]	0.534	54	0.028
Organic matter	FMA	4	0.585 [−0.028; 1.197]	0.061	11	0.355
	Malate	6	0.294 [−0.950; 1.537]	0.643	86	<0.001
	Overall	10	0.359 [−0.341; 1.059]	0.314	73	<0.001
Acid detergent fiber	FMA	2	1.344 [0.448; 2.240]	0.003	20	0.085
	Malate	6	−0.255 [−0.778; 0.269]	0.340	33	0.140
	Overall *	8	0.139 [−0.519; 0.796]	0.679	67	0.004
Neutral detergent fiber	FMA	2	0.562 [−0.925; 2.049]	0.459	74	0.049
	Malate	7	−0.175 [−0.553; 0.203]	0.365	0	0.508
	Overall	9	−0.013 [−0.418; 0.392]	0.950	30	0.166
Crude protein	FMA	4	0.797 [−0.351; 1.945]	0.174	72	0.021
	Malate	6	0.083 [−0.506; 0.673]	0.781	47	0.091
	Overall	10	0.348 [−0.216; 0.913]	0.226	61	0.008

NC, Number of comparisons; ES, effect size; CI confidence interval; Het, heterogeneity; FMA, free malic acid; *, *p* < 0.05 for between-group differences (Q_b test; χ^2^).

**Table 4:** Summary of meta-analysis results for the effects of malic acid supplementation on lamb growth performance and carcass traits.

Variable	Subgroup	NC	ES [CI]	ES *p*-Value	I^2^	Het *p*-Value
Dry matter intake	FMA	5	0.032 [−0.456; 0.519]	0.898	0	0.268
	Malate	12	−0.007 [−0.276; 0.263]	0.961	0	0.650
	Overall	17	−0.002 [−0.234; 0.238]	0.985	0	0.606
Body weight gain	FMA	7	0.772 [−0.031; 1.575]	0.059	66	0.012
	Malate	9	0.092 [−0.225; 0.410]	0.569	0	0.601
	Overall	16	0.325 [−0.010; 0.661]	0.057	37	0.029
Feed conversion ratio	FMA	3	−0.176 [−0.816; 0.465]	0.591	0	0.903
	Malate	9	−0.169 [−0.482; 0.145]	0.291	0	0.977
	Overall	12	−0.170 [−0.451; 0.111]	0.236	0	0.997
Final body weight	FMA	7	0.753 [0.162; 1.343]	0.013	36	0.093
	Malate	11	0.061 [−0.223; 0.474]	0.657	0	0.818
	Overall *	18	0.234 [−0.008; 0.475]	0.057	0	0.184
Hot carcass weight	FMA	3	0.507 [−0.458; 1.471]	0.303	41	0.183
	Malate	6	0.025 [−0.360; 0.410]	0.899	0	0.874
	Overall	9	0.106 [−0.232; 0.443]	0.540	0	0.654
Carcass yield	FMA	3	−0.075 [−0.977; 0.828]	0.871	36	0.189
	Malate	6	−0.123 [−0.508; 0.263]	0.533	0	0.892
	Overall	9	−0.124 [−0.461; 0.213]	0.470	0	0.757

NC, Number of comparisons; ES, effect size; CI confidence interval; Het, heterogeneity; FMA, free malic acid; *, *p* < 0.05 for between-group differences (Q_b test; χ^2^).

**Table 5:** Meta-regression of dietary moderators (g/kg BW) and forage proportion ^1^ on performance, ruminal parameters and nutrient digestibility of lambs.

Variables	NC	CP		NDF		ADF		Starch		Forage		Concentrate		Forage Proportion	
		Slope	R^2^	Slope	R^2^	Slope	R^2^	Slope	R^2^	Slope	R^2^	Slope	R^2^	Slope	R^2^
Performance															
BWG	16	−0.064	34.03	0.013	0.00	0.042	0.00	−0.19	0.00	0.071 *	46.56	−0.018 *	59.88	3.863 **	87.44
Ruminal parameters															
NH_3_-N	9	0.157	0.00	−0.063	0.00	−0.096	0.00	0.025	0.00	−0.112 **	97.82	0.042	39.13	−4.816 **	100.00
Acetate	6	0.086	0.00	−0.030	0.00	−0.045	0.00	0.065	0.00	−0.080	0.00	0.033	0.00	−5.467	41.74
Propionate	6	0.098	0.00	0.201	60.85	0.272 *	100.00	−0.088	27.00	0.192 *	100.00	−0.041	0.00	6.944 *	85.19
Butyrate	6	0.341	100.00	−0.022	0.00	−0.059	0.00	0.098	100.00	−0.068	0.00	0.052	100.00	−1.887	0.00
Total VFAC	10	−0.221	21.07	0.079	72.38	0.073	22.17	−0.029	0.00	0.066 *	100.00	−0.038	68.42	2.295 *	83.08
Nutrient digestibility															
DM	9	−0.074	0.00	0.036	0.00	0.020	0.00	−0.018	0.00	0.045	18.34	−0.014	0.00	1.645	12.41
OM	10	−0.219	55.21	0.239 *	43.11	0.276 *	60.64	−0.086	42.98	0.082	35.18	−0.037 *	53.53	3.061	24.19
ADF	8	−0.282 *	100.00	0.111	11.88	0.097	4.45	−0.099	58.79	0.085 *	77.20	−0.041 *	98.54	3.936 *	84.96
NDF	9	−0.077	0.00	0.102	100.00	0.114	100.00	−0.037	0.00	0.053 *	100.00	−0.016	13.42	2.300 *	100.00
CP	10	−0.127	10.03	0.033	0.00	0.030	0.00	−0.033	0.00	0.066	30.99	−0.025	22.87	2.703	28.67

CP, Crude Protein; NDF, Neutral detergent fiber; ADF, Acid detergent fiber; BWG, Body weight gain; NH_3_-N, ammoniacal nitrogen; VFAC, Volatile fatty acids concentration; DM, Dry matter, OM, organic matter; Acetate, butyrate and propionate are expressed as proportion; ^1^ Forage proportion was calculated as forage/(forage + concentrate), based on the actual intake values reported in the studies. * *p* < 0.05 and ** *p* < 0.01 by the *t*-test for the regression coefficient (slope).

## Data Availability

No new data were created or analyzed in this study. All data supporting the findings are publicly available in the original publications referenced in the manuscript.

## Supplementary Materials

The following supporting information can be downloaded at: https://www.mdpi.com/article/10.3390/ani16081263/s1, Figure S1: Study-level risk-of-bias judgments within each bias domain for the 12 studies evaluating the effects of malic acid in lambs. Figure S2: Leave-one-out analysis for ruminal parameters of lambs supplemented with malic acid. Figure S3: Funnel plot of the effect of malic acid on ruminal parameters of lambs for assessing publication bias. Figure S4: Leave-one-out analysis for ruminal volatile fatty acids concentration of lambs supplemented with malic acid. Figure S5: Funnel plot of the effect of malic acid on ruminal volatile fatty acids concentration of lambs for assessing publication bias. Figure S6: Leave-one-out analysis for ruminal volatile fatty acids proportion of lambs supplemented with malic acid. Figure S7: Funnel plot of the effect of malic acid on ruminal volatile fatty acids proportion of lambs for assessing publication bias. Figure S8: Leave-one-out analysis for nutrient digestibility of lambs supplemented with malic acid. Figure S9: Funnel plot of the effect of malic acid on nutrient digestibility of lambs for assessing publication bias. The vertical line represents the overall effect size estimate. Figure S10: Leave-one-out analysis for growth performance of lambs supplemented with malic acid. Figure S11: Funnel plot of the effect of malic acid on growth performance of lambs for assessing publication bias. Figure S12: Leave-one-out analysis for carcass characteristics of lambs supplemented with malic acid. Figure S13. Funnel plot of the effect of malic acid on carcass characteristics of lambs for assessing publication bias.
