# Supplementary material for: Malic Acid Supplementation on Rumen Fermentation, Nutrient Digestibility, Performance and Carcass Traits in Lambs: A Meta-Analysis and Meta-Regression Considering Dietary Moderators
Source: Animals (Basel). 2026 Apr 20;16(8):1263. doi: 10.3390/ani16081263 (PMC13113034; doi:10.3390/ani16081263)
Supplement: Supplementary file 1 [file animals-16-01263-s001.zip › animals-4226544-supplementary.pdf]

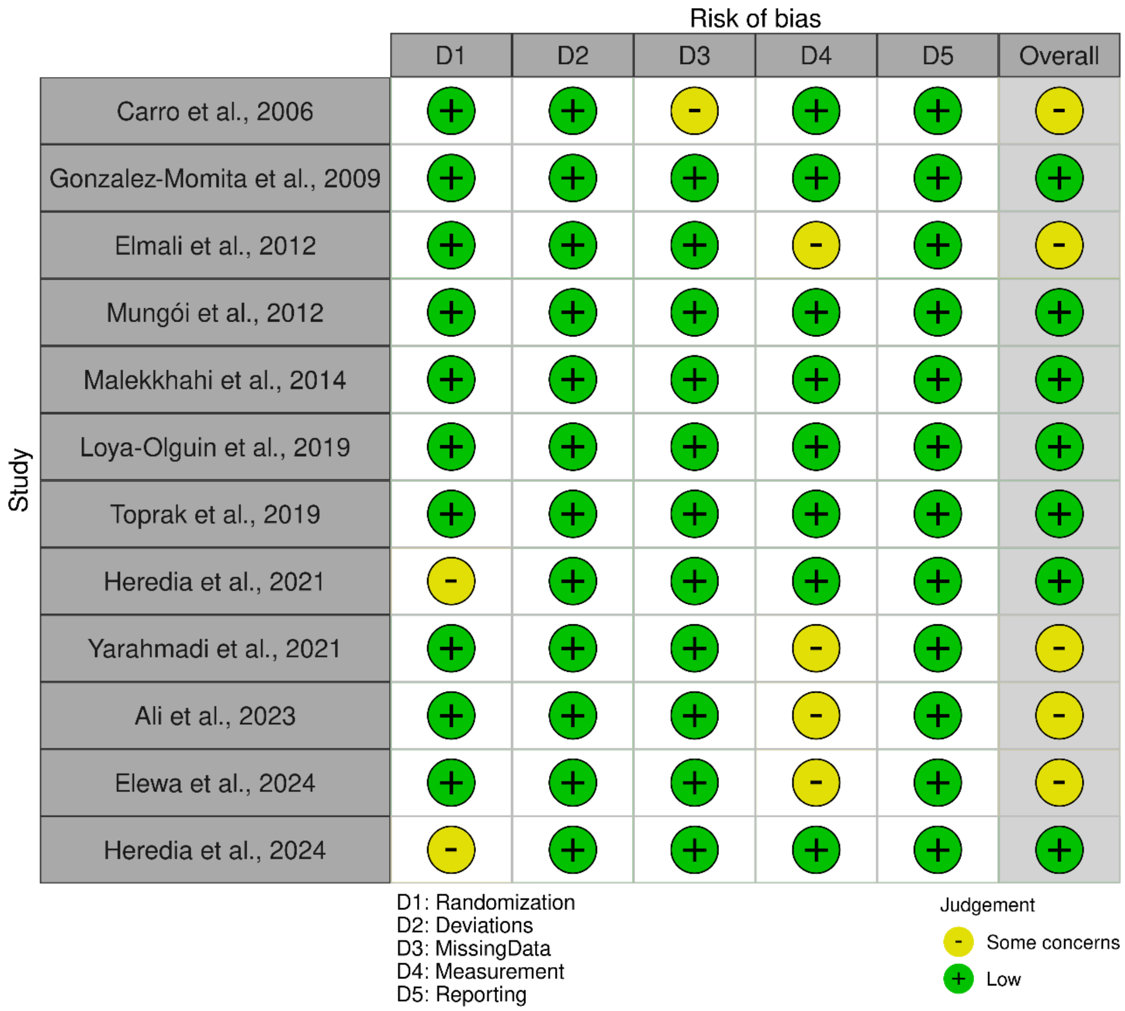

Figure S1. Study-level risk-of-bias judgments within each bias domain for the 12 studies evaluating the effects of malic acid in lambs.

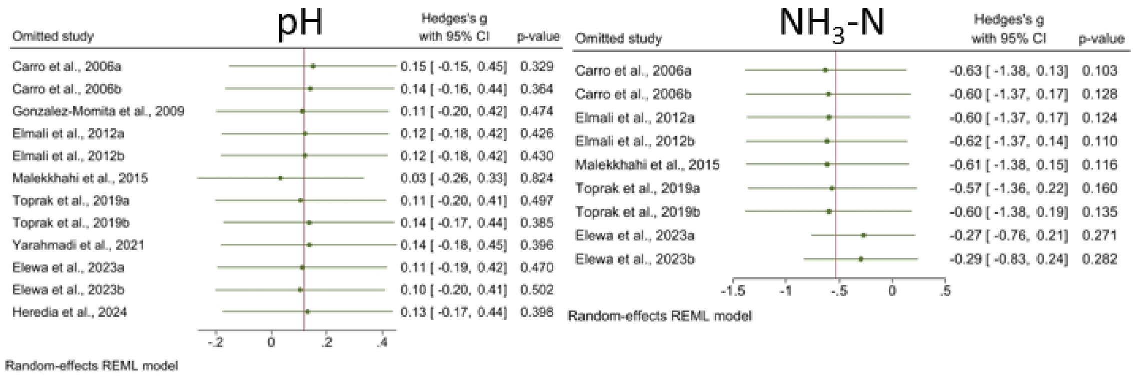

Ammoniacal nitrogen - NH_3-N

Figure S2: Leave-one-out analysis for ruminal parameters of lambs supplemented with malic acid. The plot depicts the impact of removing each individual study on the point estimate of the effect size, as well as on the lower and upper 95% confidence intervals (CI).

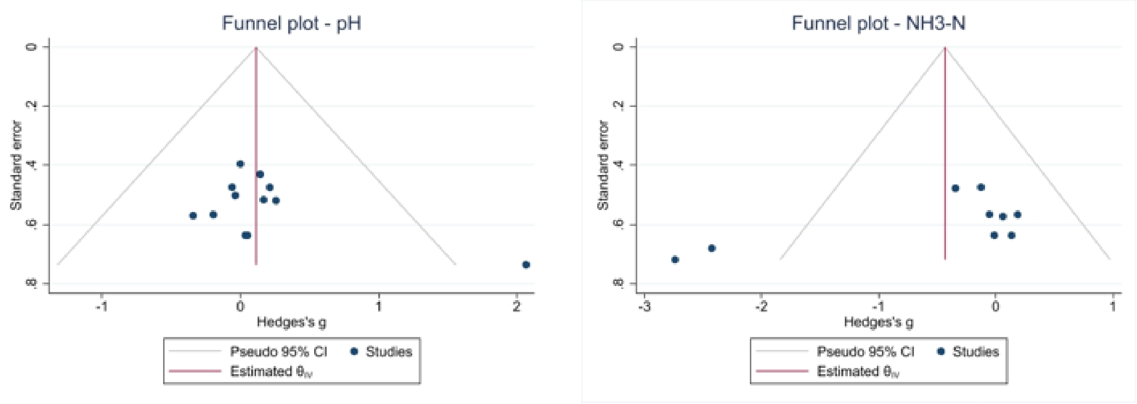

Ammoniacal nitrogen - NH_3-N

Figure S3: Funnel plot of the effect of malic acid on ruminal parameters of lambs for assessing publication bias. The vertical line represents the overall effect size estimate. The two diagonal lines indicate the 95% confidence interval around the effect size estimate. Publication bias may be present if an unequal number of studies, particularly smaller studies, are distributed on one side of the vertical line.

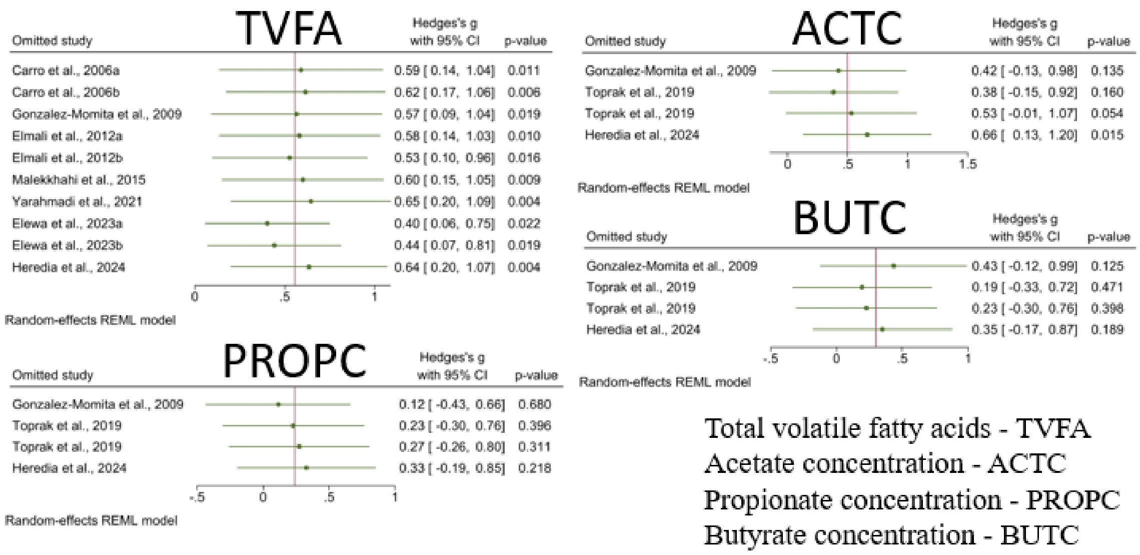

Figure S4: Leave-one-out analysis for ruminal volatile fatty acids concentration of lambs supplemented with malic acid. The plot depicts the impact of removing each individual study on the point estimate of the effect size, as well as on the lower and upper 95% confidence intervals (CI).

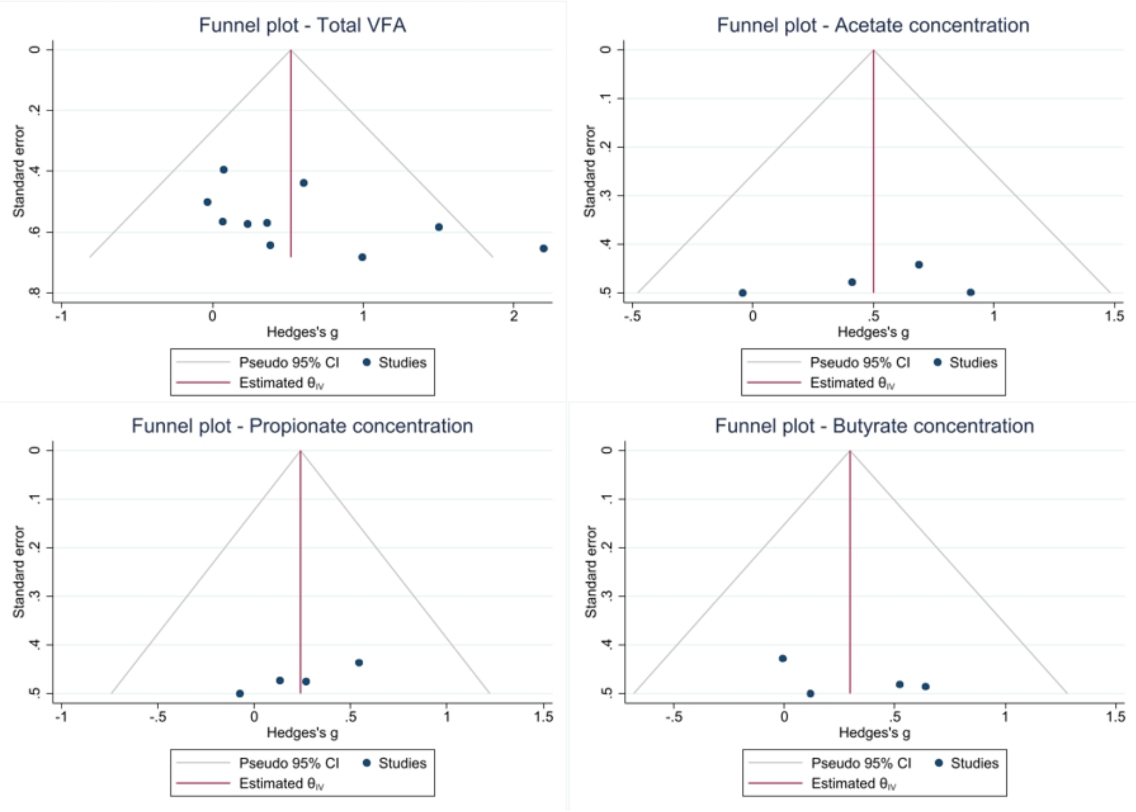

Figure S5: Funnel plot of the effect of malic acid on ruminal volatile fatty acids concentration of lambs for assessing publication bias. The vertical line represents the overall effect size estimate. The two diagonal lines indicate the 95% confidence interval around the effect size estimate. Publication bias may be present if an unequal number of studies, particularly smaller studies, are distributed on one side of the vertical line.

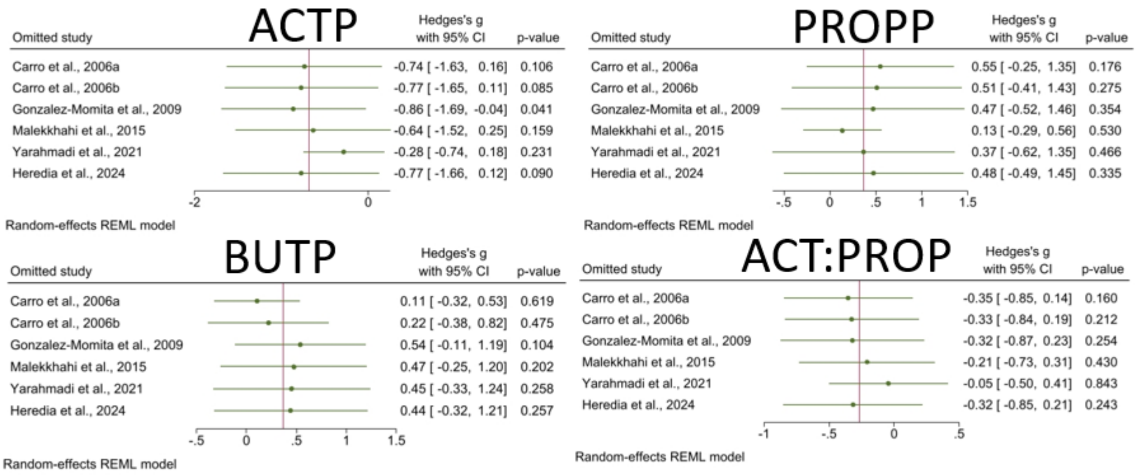

Acetate to propionate ratio - ACP:PROP
Acetate proportion - ACTP
Propionate proportion - PROPP
Butyrate proportion - BUTP

Figure S6: Leave-one-out analysis for ruminal volatile fatty acids proportion of lambs supplemented with malic acid. The plot depicts the impact of removing each individual study on the point estimate of the effect size, as well as on the lower and upper 95% confidence intervals (CI).

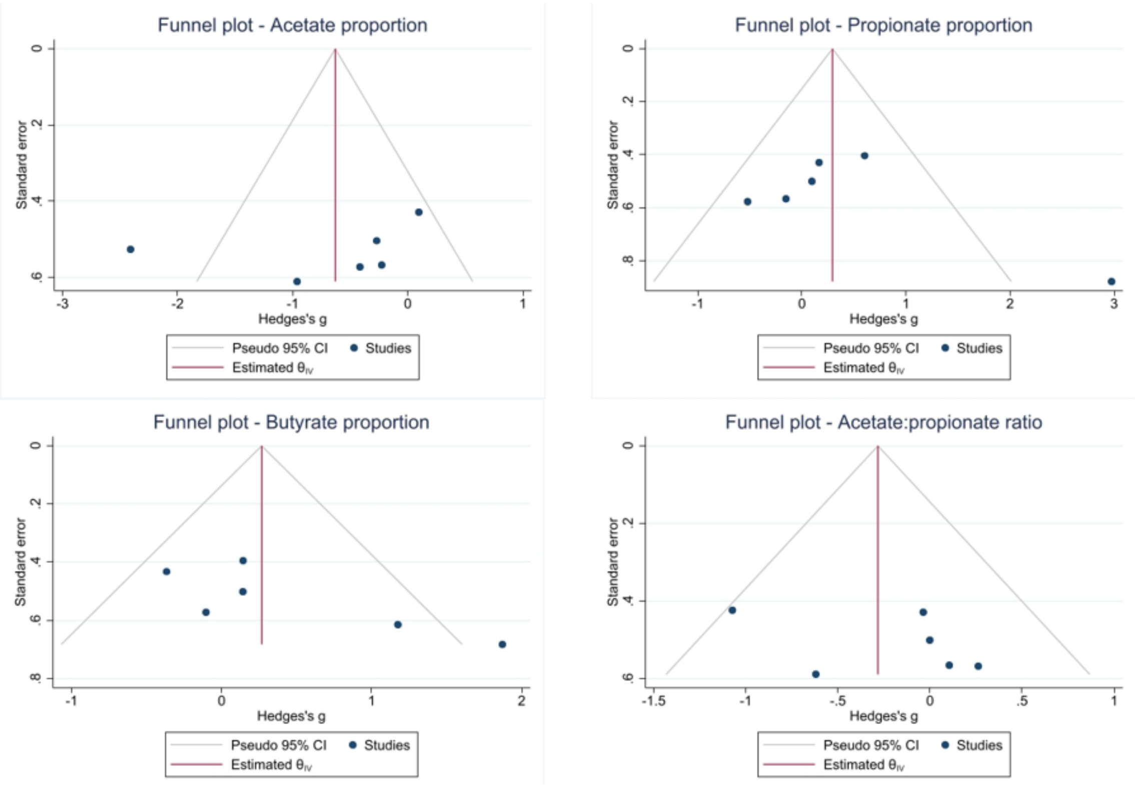

Figure S7: Funnel plot of the effect of malic acid on ruminal volatile fatty acids proportion of lambs for assessing publication bias. The vertical line represents the overall effect size estimate. The two diagonal lines indicate the 95% confidence interval around the effect size estimate. Publication bias may be present if an unequal number of studies, particularly smaller studies, are distributed on one side of the vertical line.

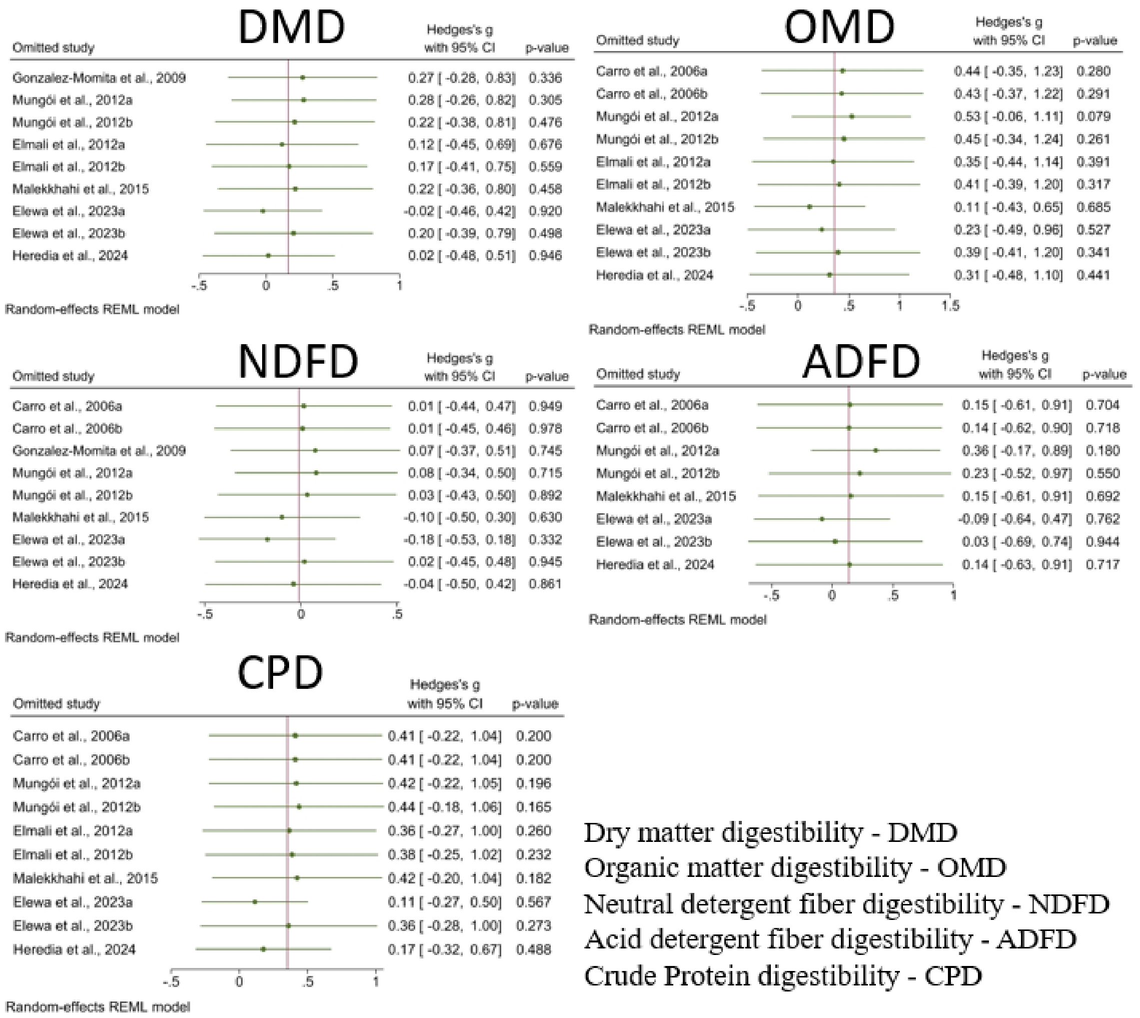

Figure S8: Leave-one-out analysis for nutrient digestibility of lambs supplemented with malic acid. The plot depicts the impact of removing each individual study on the point estimate of the effect size, as well as on the lower and upper 95% confidence intervals (CI).

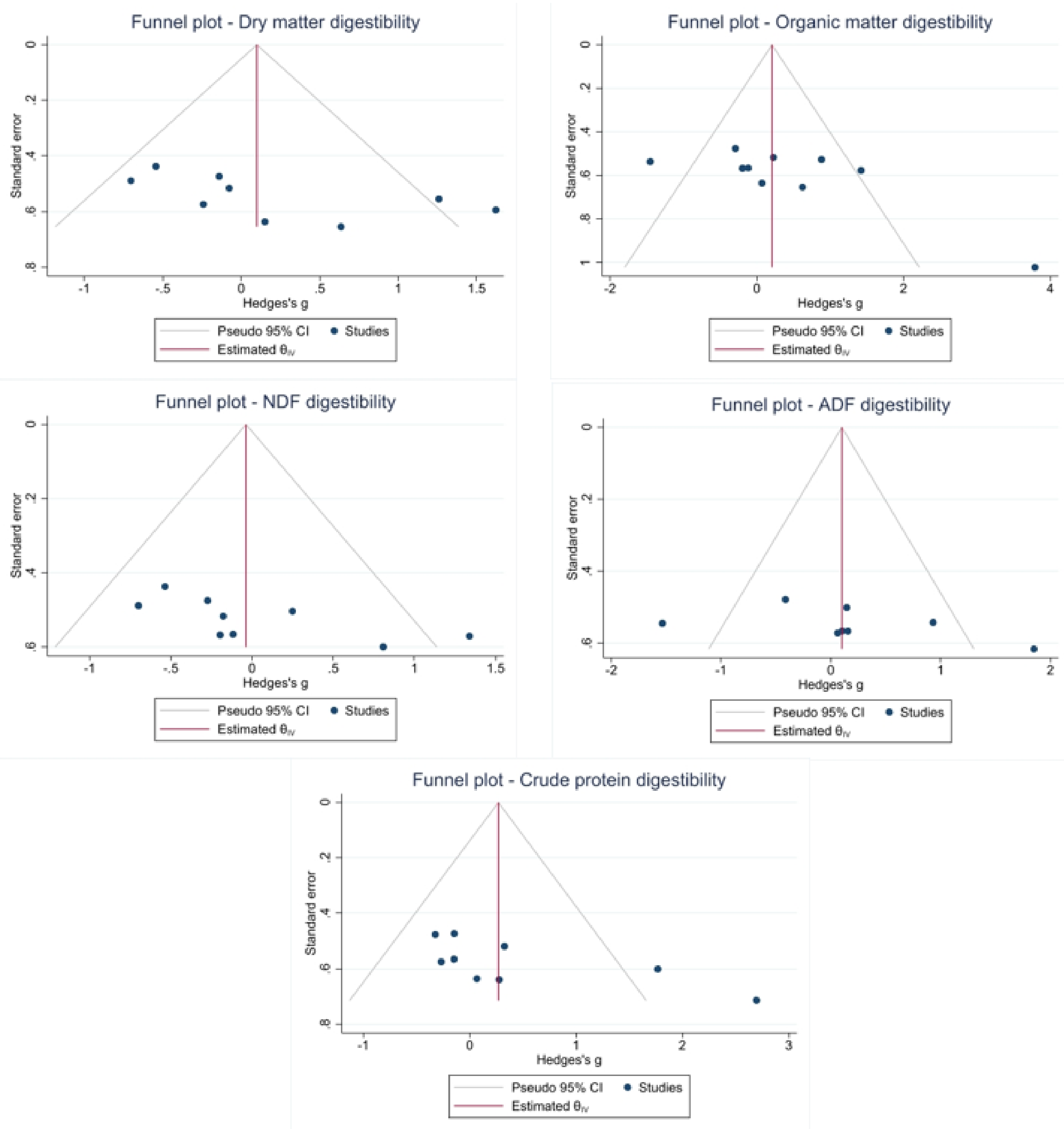

Figure S9: Funnel plot of the effect of malic acid on nutrient digestibility of lambs for assessing publication bias. The vertical line represents the overall effect size estimate. The two diagonal lines indicate the 95% confidence interval around the effect size estimate. Publication bias may be present if an unequal number of studies, particularly smaller studies, are distributed on one side of the vertical line.

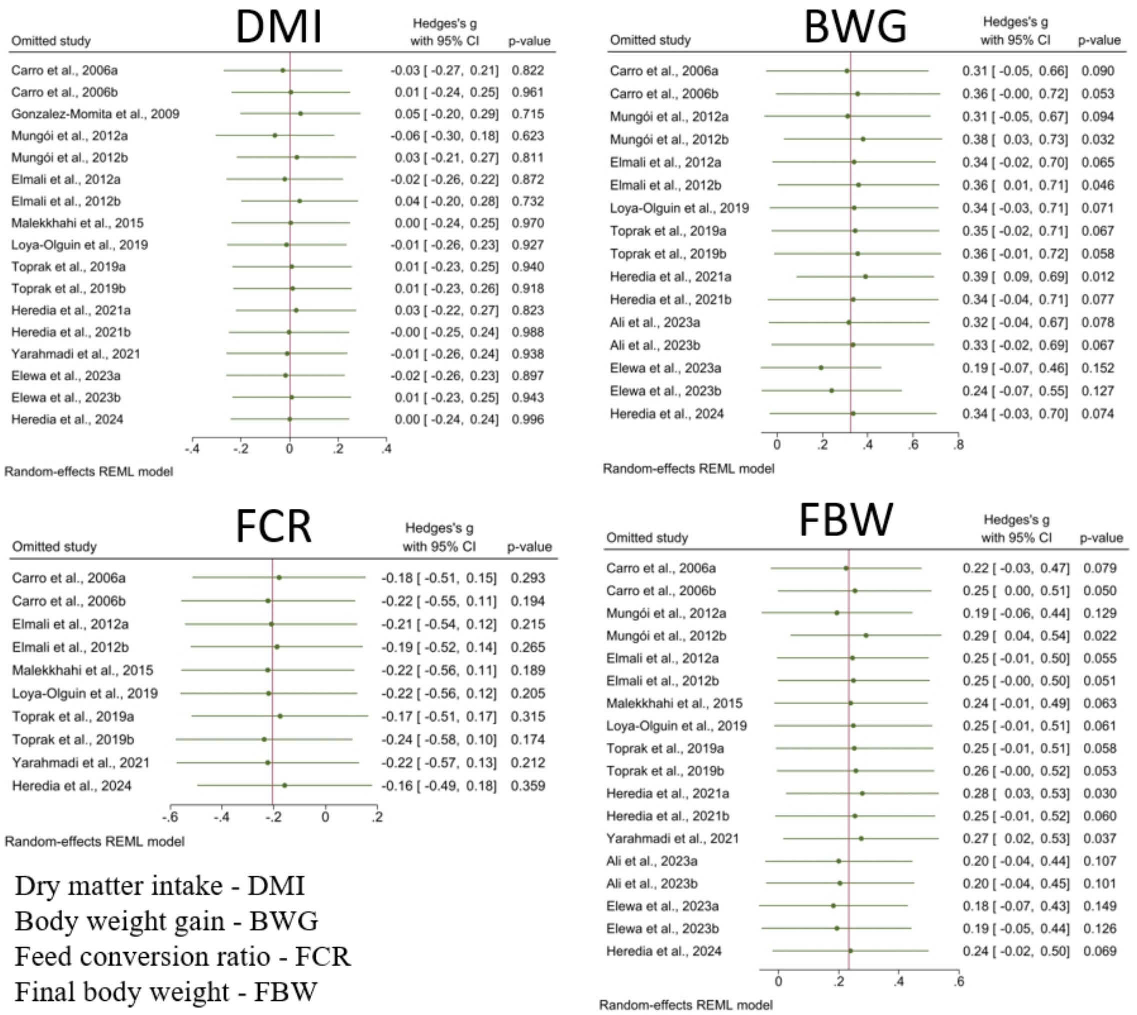

Figure S10: Leave-one-out analysis for growth performance of lambs supplemented with malic acid. The plot depicts the impact of removing each individual study on the point estimate of the effect size, as well as on the lower and upper 95% confidence intervals (CI).

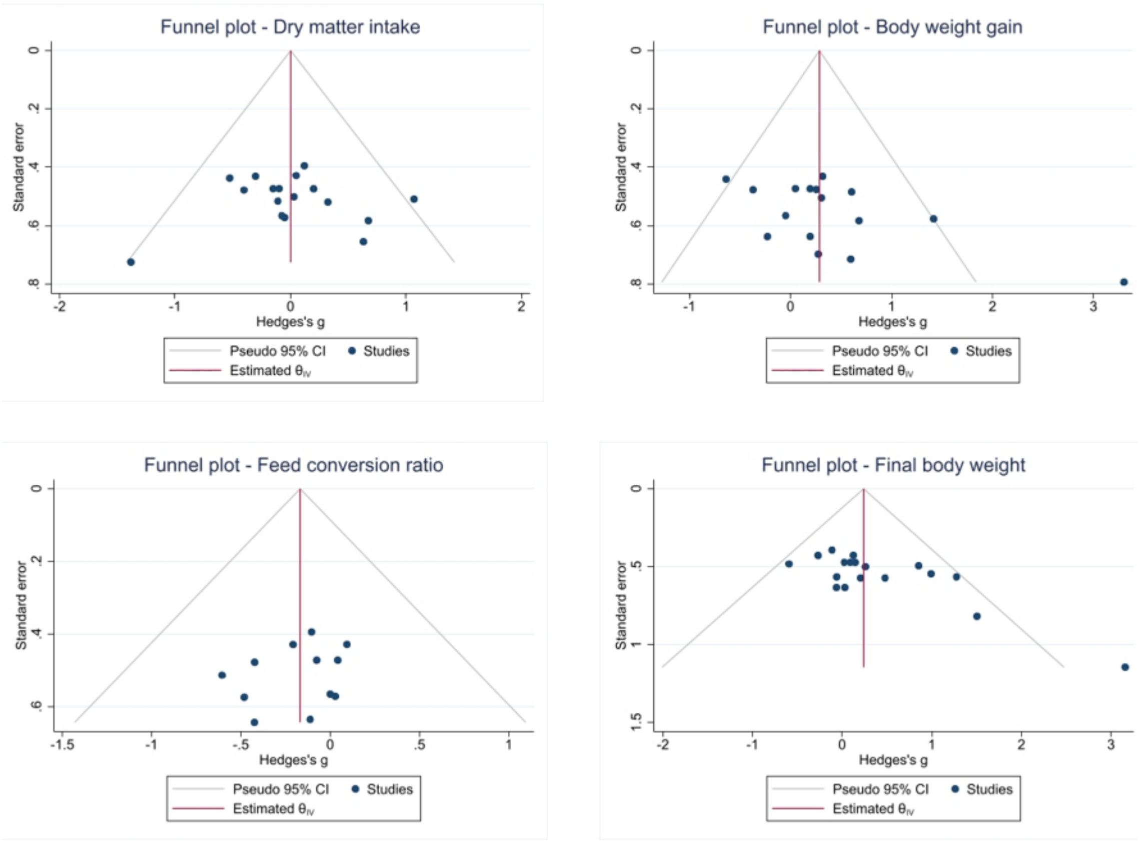

Figure S11: Funnel plot of the effect of malic acid on growth performance of lambs for assessing publication bias. The vertical line represents the overall effect size estimate. The two diagonal lines indicate the 95% confidence interval around the effect size estimate. Publication bias may be present if an unequal number of studies, particularly smaller studies, are distributed on one side of the vertical line.

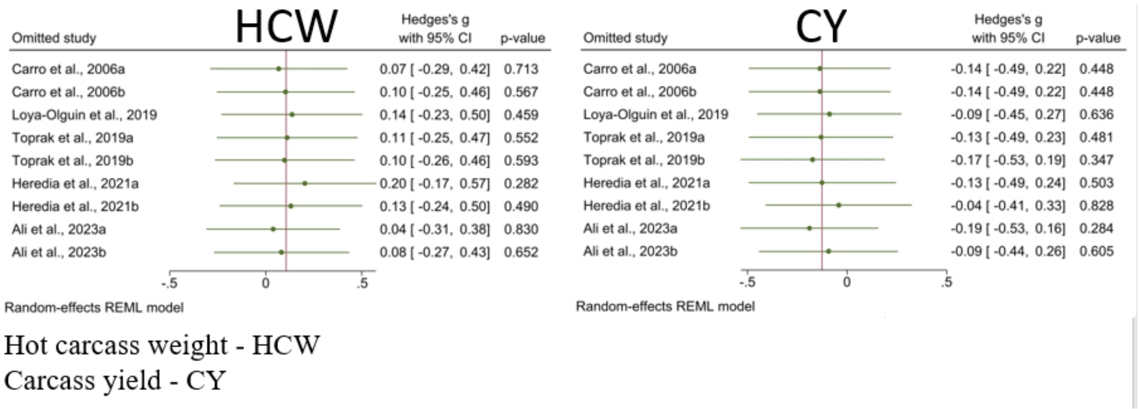

Figure S12: Leave-one-out analysis for carcass characteristics of lambs supplemented with malic acid. The plot depicts the impact of removing each individual study on the point estimate of the effect size, as well as on the lower and upper 95% confidence intervals (CI).

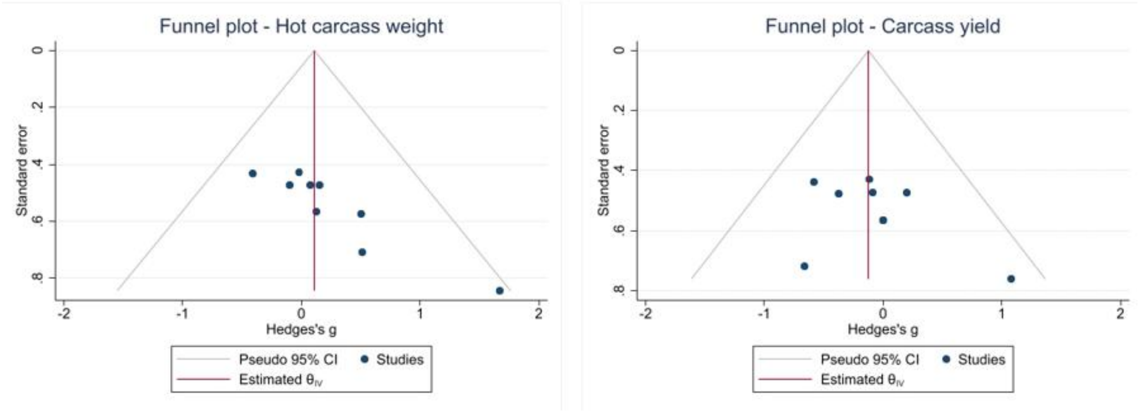

Figure S13. Funnel plot of the effect of malic acid on carcass characteristics of lambs for assessing publication bias. The vertical line represents the overall effect size estimate. The two diagonal lines indicate the 95% confidence interval around the effect size estimate. Publication bias may be present if an unequal number of studies, particularly smaller studies, are distributed on one side of the vertical line.
